# Versatile *Lactococcus lactis* strains improve texture in both fermented milk and soybean matrices

**DOI:** 10.1093/femsle/fnac117

**Published:** 2022-12-01

**Authors:** Vera Kuzina Poulsen, Elahe Ghanei Moghadam, Stjepan Krešimir Kračun, Birgit Albrecht Svendsen, Wioleta Marta Nielsen, Gunnar Oregaard, Anders Krarup

**Affiliations:** Discovery R&D, Chr. Hansen A/S, 10–12 Bøge Allé, DK-2970 Hørsholm, Denmark; Discovery R&D, Chr. Hansen A/S, 10–12 Bøge Allé, DK-2970 Hørsholm, Denmark; Discovery R&D, Chr. Hansen A/S, 10–12 Bøge Allé, DK-2970 Hørsholm, Denmark; Discovery R&D, Chr. Hansen A/S, 10–12 Bøge Allé, DK-2970 Hørsholm, Denmark; Discovery R&D, Chr. Hansen A/S, 10–12 Bøge Allé, DK-2970 Hørsholm, Denmark; Discovery R&D, Chr. Hansen A/S, 10–12 Bøge Allé, DK-2970 Hørsholm, Denmark; Discovery R&D, Chr. Hansen A/S, 10–12 Bøge Allé, DK-2970 Hørsholm, Denmark

**Keywords:** polysaccharides, texture, *Lactococcus lactis*

## Abstract

Lactic acid bacteria (LAB) have long been used to extend the shelf life and improve the taste and texture of fermented milk. In this study, we investigated the texturing potential of LAB in plant-based fermentation by high-throughput screening of 1232 *Lactococcus lactis* strains for texture in milk and liquid soybean matrices. We found that most strains with texturing abilities in fermented milk were also capable of enhancing the texture in fermented soybean, despite the large differences in composition of the two matrices. Exocellular polysaccharide production is believed to contribute positively to fermented milk and plant-base texture. It appeared as if it was the properties of the polysaccharides rather than their protein interaction partners that were responsible for the enhanced texture in both matrices. We mined whole genome sequences of texturing strains for polysaccharide biosynthesis (*eps*) gene clusters. The comparative genomics approach revealed 10 texturing strains with novel *eps* gene clusters. Currently, the relationship between the novel genes and their functionality in milk and plant matrices is unknown.

## Teaser

Texturing lactic acid bacteria in fermented milk were also texturing in fermented soybean, which is related to the properties of polysaccharides; novel genes involved in the texture formation were identified.

## Introduction

Many food consumers request plant-based alternatives to conventional dairy and meat products for reasons such as sustainability, lifestyle, and dietary restrictions. The texture of fermented milk or plant-based alternatives is an important quality parameter, as it affects consumer acceptance. Fermentation can aid in improving the sensory profiles, nutritional properties, texture, and microbial safety of plant-based dairy and meat alternatives, thereby possibly eliminating the use of flavor masking and texturing ingredients.

Even though plant-based milk substitutes may have many compositional and structural similarities to cow’s milk, the aroma, taste, and texture of plant-based milk substitutes are different because they contain different types as well as different ratios of protein, carbohydrate, and fat. Today, most starter cultures, which are key for the fermentation process, and improve the quality of the end products, have been developed to ferment milk products. As the matrix plays an important part in the fermentation process, the application of a dairy culture in a plant-based matrix might not be optimal due to the differences in matrix composition. Thus, there is an opportunity to look for strains that produce better fermentation results in plant bases and develop these into efficient starter cultures, which achieve the desired dairy product characteristics in plant-based substrates.

Polysaccharide-producing bacteria can positively influence fermented milk product characteristics such as texture, water-holding capacity, and sensory properties (Mende et al. 2016, Zeidan et al. 2017, Surber et al. 2021). The most widespread pathway for exopolysaccharide production is known as the Wzy-dependent pathway, which is found in Gram-positive and Gram-negative bacteria. Polysaccharides generated through this pathway include some of the largest glycopolymers (molecular weight > 10^6^ Da) and can have useful viscosifying and pseudoplastic properties of commercial value (Whitfield et al. 2020). Genes encoding Wzy-dependent exocellular polysaccharide biosynthesis proteins in Lactic acid bacteria (LAB) are organized in a cluster with an operon structure. Both the *eps* gene clusters and the structures of the polysaccharides are very diverse (Zeidan et al. 2017). These polymers are very heterogenous and can consist of a single or multiple monosaccharide building blocks, they can be unbranched or branched, and neutral or charged. This polysaccharide heterogeneity, in terms of localization, composition, charge, and size, influences the textural properties (Zeidan et al. 2017). The overall charge of the polysaccharides, together with the amount produced during fermentation, determines the distribution of the polysaccharides in the microstructure, and hence their interference with gelation and interaction with the protein matrix. The majority of exocellular polysaccharides produced by LAB are uncharged. In milk, a few reported negatively charged polysaccharides, e.g. anionic polysaccharides from *Lactococcus lactis* ssp. *cremoris* NIZO B40 strain (NCC2771), have been associated with higher viscoelastic properties, stiffness, viscosity, and high shear resistance as a result of ionic bond formation (Girard and Schaffer-Lequart 2008, Gentès et al. 2011, Kristo et al. 2011). In plant-based foods, polysaccharides are often used as functional ingredients to increase viscosity (Xu et al. 2018, McClements and Grossmann 2021). The thickening power of a polysaccharide increases with increased molecular weight, reduced branching, and with spatially elongated conformations. For this reason, stiff elongated polysaccharides (such as xanthan gum) have a much higher thickening power than spatially compact globular polysaccharides (such as gum arabic) (McClements and Grossmann 2021).

Milk proteins, e.g. caseins, have a molecular weight of 19–24 kDa, while β-lactoglobulin and α-lactalbumin are 18.3 kDa and 14.2 kDa, respectively. The milk proteins are small compared to the major plant proteins, e.g. β-conglycinin and glycinin in soybean have molecular weights of 150–200 kDa and 300–380 kDa, respectively (Alves and Tavares 2019) and form larger macromolecular complexes containing numerous proteins. The most common plant proteins currently used in plant-based foods include soy, pea, potato, mung bean, and rice proteins (Sha and Xiong 2020). Furthermore, milk proteins are relatively flexible and quite disordered structures and contain numerous anionic phosphate groups, enabling cross-linking by calcium ions, which is a property crucial for their ability to form gels. Caseins have a highly flexible almost random coiled structure, with hydrophobic and hydrophilic patches and numerous phosphate groups forming a micelle-like structure, which in yogurt and cheese gives rise to their unique textural characteristics. Additionally, they form tight globular structures, which plant proteins do not. Many of the proteins isolated from plant sources are relatively large globular storage proteins that are naturally found as part of supramolecular structures (McClements and Grossmann 2021).

Interactions of exopolysaccharides and proteins are of great importance for texture development in milk (Hassan 2008, Corredig et al. 2011, Mende et al. 2016, Birch et al. 2017, 2021) and plant-based foods (Xu et al. 2019, Grossmann and McClements 2021, McClements and Grossmann 2021, Yang et al. 2021). However, the mechanism of interaction remains largely unknown, mainly due to limitations in the identification and visualization of the polysaccharides.

Most milk analogs are colloidal dispersions consisting of oil bodies, fat droplets, protein aggregates, and plant tissue fragments suspended in an aqueous solution (McClements and Grossmann 2021). Biopolymers with exposed nonpolar regions on their surfaces, such as many plant-based proteins, can bind nonpolar molecules through hydrophobic interactions. Milk analogs with high viscosities often contain thickening agents to inhibit gravitational separation of large particles and to provide a creamy mouthfeel due to the lack of oil bodies and fat droplets. Plant-derived polysaccharides such as pectin, locust bean gum, gellan gum, starch, methylcellulose, and algal polysaccharides such as carrageenan and alginate are often used as thickening agents or stabilizers in food matrices (Phillips and Williams 2021). The nature of the interactions between the colloidal particles in milk analogs most likely depends on their specific composition and the mechanism is largely unknown (Sandoval Murillo et al. 2019, Weiss et al. 2019).

*Lactococcus lactis* is used to produce numerous fermented dairy products including cheese and mesophilic fermented milk, such as buttermilk and sour cream. The aim of this work was to identify texturing *L. lactis* strains in plant bases with high-throughput texture screening of fermented soybean using a collection of 1232 *L. lactis* strains and compare their performance with regard to texture in milk. Moreover, we investigated the texturing strains for the presence of novel *eps* gene clusters.

## Materials and methods

A total of 1232 *L. lactis* strains originating from the Chr. Hansen culture collection were screened for the ability to enhance texture in fermented milk or soybean matrices using TADM (total aspiration dispense monitoring) in 2-ml microtiter plates as described previously (Poulsen et al. 2019). Heat-treated skimmed milk (Havmand et al. 2022) added 0.2% w/v yeast extract (NuCel® 545 MG, batch number 0005115910, batch AD 18 A05030, Procelys) or a ready-made soybean drink (Naturli’ Økologisk Soyadrik, Naturli’, Naturli’ Foods, Vejen, Denmark) supplemented with 2% w/v glucose were used. Only a few *L. lactis* strains were able to ferment the soybean matrix, which contained 0.1 g carbohydrate per 100 g according to the nutritional facts, unless additional glucose was added. Several *L. lactis* strains would not be able to ferment milk, unless yeast extract, which provides *L. lactis* with peptides, is added. The texturing properties of selected strains were validated in 200-ml scale using rheometry as described previously (Poulsen et al. 2019). Soybean and milk matrices were added 1% v/v of an overnight inoculum, and incubated at 30°C until pH∼4.55, and then cooled to 4°C. The samples were subsequently stirred gently and used to measure the shear stress at different shear rates as described previously (Poulsen et al. 2019). Heat-treated skimmed milk with the shear stress of above 35 Pa at a shear rate 300/s, as a result of fermentation, was considered as having a good texture.

The texture of five selected strains grown in milk and soybean base with and without degraded protein was conducted by pregrowing the strains in the M17-K medium with 2% w/v glucose at 30°C overnight in 2-ml microtiter plates. After pregrowth, the strains were inoculated into either milk or soybean drink supplemented with 2% w/v glucose and a pH indicator as previously described (Poulsen et al. 2019). The inoculum size into the matrices was 1% v/v. In parallel, before inoculation, a proteinase K (Sigma-Aldrich) solution (final specific activity 4 U/ml) in 50 mM Tris-HCl buffer pH 7.0 with 1 mM CaCl_2_ (protease buffer) was added to the respective bases at 2.5% v/v ratio. As a buffer control, the protease buffer was added to bases at the same v/v ratio but without proteinase K. The fermentations in the presence or absence of proteinase K and the protease buffer were grown overnight (18 h) at 30°C, while monitoring the pH every 6 min as described previously (Poulsen et al. 2019) and then cooled to 4°C.

The novel *eps* gene cluster sequences obtained from proprietary strains were deposited in GenBank under the following accession numbers: LC-04, ON023532; LC-05, ON023527; LC-06, ON023530; LC-07, ON023529; LC-08, ON023534; LC-09, ON023531; LC-12, ON023528; LC-13, ON023533; LC-14, ON023535; and LC-20, ON023536. The degree of sequence identity was determined using the multiple sequence alignment tool Clustal Omega (https://www.ebi.ac.uk/Tools/msa/clustalo/) with standard parameters. DNA extraction, genome sequencing, and mining for the *eps* gene clusters were performed as described previously (Poulsen et al. 2020).

## Results and discussion

### High-throughput screening for texturing strains

**Figure 1.**
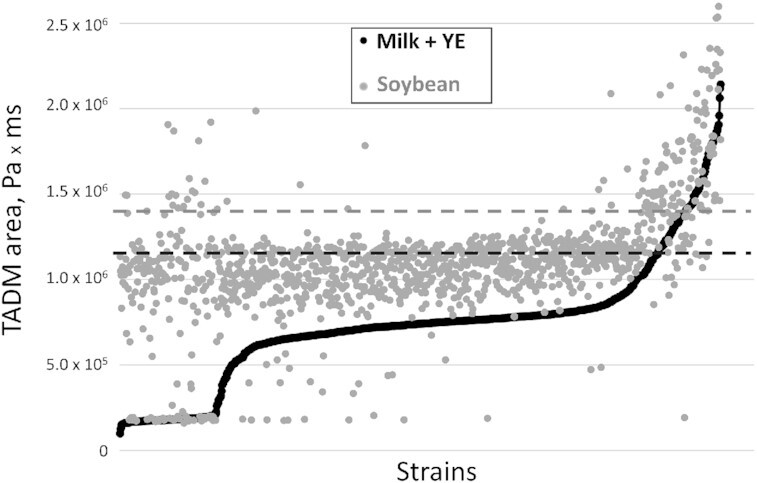
Texture (TADM area) of 1232 soybean or milk with yeast extract samples fermented with different *L. lactis* strains in 2-ml microtiter plate scale. The dotted gray line indicates the threshold for what we consider texturing strains in soybean, while the black dotted line indicates the corresponding threshold in milk-with-added-yeast-extract (Milk + YE).

**Figure 2.**
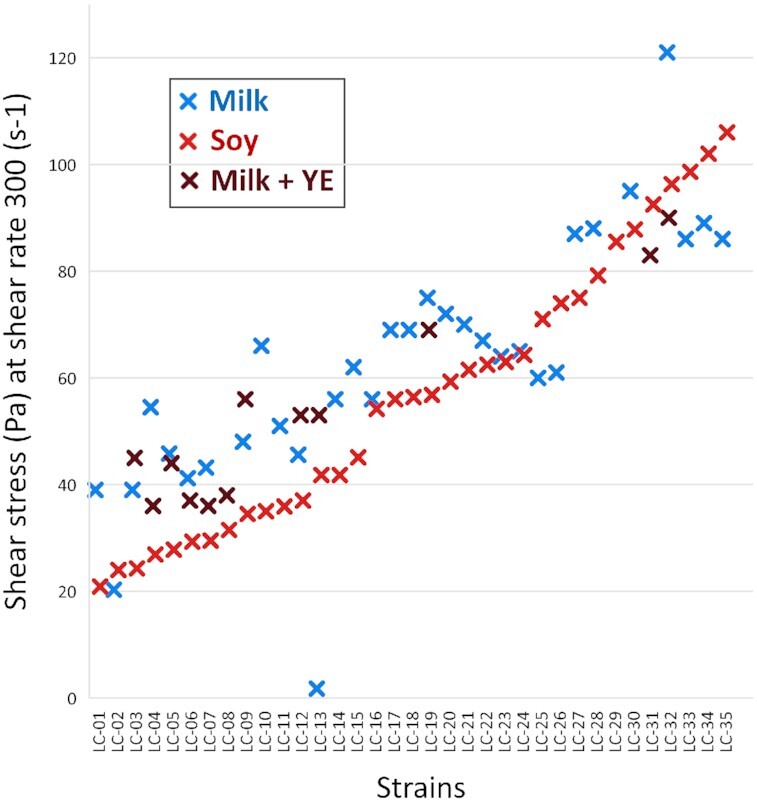
Texture (shear stress) of soybean and milk ± yeast extract (YE) samples fermented with selected *L. lactis* strains in 200-ml scale at 30°C.

Milk and soybean matrices individually fermented with 1232 *L. lactis* strains were screened for texture using the Hamilton Microlab Star liquid handler, which was used to collect pressure versus time data using TADM tool. TADM aspiration pressure curves were obtained by aspirating the samples using Hamilton liquid robot and used as proxy for shear stress measurements, to identify samples with elevated texture as in Poulsen et al. (2019). Strains were considered texturing when giving rise to elevated texture in fermented milk samples (TADM area ≥ 1.2 million Pa × ms) or fermented soybean samples (TADM area ≥ 1.4 million Pa × ms; Fig. 1). Based on these thresholds, 69 strains were considered texturing in soybean and 46, in milk added yeast extract. A total of 33 strains were texturing in both milk containing yeast extract and soybean. That is, almost half of the texturing leads in soybean were also considered texturing in milk containing yeast extract, while 72% of the texturing leads in milk with yeast extract were also considered texturing in soybean. The large overlap of the strains being ranked as texturing in both milk with yeast extract and soybean, despite the many differences between the matrices, indicates that texture is more dependent on the texturing capabilities of strains rather than the matrix composition. The leads from the microtiter plate experiments were confirmed to produce good texture in fermentations conducted in 200-ml scale, where milk and soybean acidification was monitored using pH electrodes and stopped at the pH∼4.55 by transferring the samples to an ice water bath, followed by rheology measurements. Some of the strains under investigation, e.g. LC-01, were slow acidifiers and that may be due to low proteolytic activity, since in the absence of proteases, the nitrogen and carbon acquisition through protein catabolism is severely limited thus impeding growth and subsequent acidification. The slow acidification can be circumvented by the addition of peptides, e.g. caseinate or yeast extract. Alternatively, coinoculation with a helper strain may be used for increasing the proteolytic power required for the release of peptides from the protein source in the fermented base. Thus, LC-01 was not texturing in milk, as the milk did not coagulate after overnight incubation, while it was texturing in milk with yeast extract and in soybean matrices (Fig. 2).

Some of the strains that were found texturing in soybean were not able to grow in milk with yeast extract, likely because of their inability to utilize lactose as a sole carbon source. The average pH of the strains with the TADM area below 2.0 × 10^5^ in milk with yeast extract was 5.6, while it was 4.4 for the same strains in soybean. The average pH of the strains with the TADM area above 2.0 × 10^5^ in both milk with yeast extract and soybean after an overnight incubation was 4.2. The relatively high pH of the strains with the TADM area below 2.0 × 10^5^ in milk added yeast extract can likely be explained by their poor ability to grow on lactose as the sole carbon source. When grown in M17-K added 2% lactose, their average OD_620_ was 0.25, while it was 0.87 when grown in glucose as the sole carbon source. Strains with the TADM area above 2.0 × 10^5^ in milk with yeast extract, which were able to acidify milk, had an average OD_620_ of 0.96 in M17-K added 2% lactose, while it was 1.02 when grown in M17-K added 2% glucose.

LC-02 was nontexturing in the matrices tested. Several strains that can be considered texturing in milk were evidently nontexturing in soybean, e.g. LC-01, LC-03, LC-04, LC-05, LC-06, and LC-07 (Fig. 2). However, it should be noted that the thresholds in milk were established based on many application trials in production scale (Poulsen et al. 2019), while only a very limited number of application trials have been performed using soybean. Thus, setting a texture threshold for soybean based on application trials was not possible due to the lack of prior art, and therefore, we here have used the threshold established for milk: shear stress above 35 Pa at shear rate 300/s.

When milk was enriched with yeast extract, thereby facilitating acidification of milk by most strains, most texturing strains produced good texture in both matrices (correlation of R^2^ = 0.90; Fig. 2). In contrast, when milk was used without yeast extract, the correlation between texture in milk and soybean was smaller (R^2^ = 0.66), because some strains did not acidify milk and result in a milk gel formation with measurable texture unless yeast extract was added.

### Role of polysaccharides in the texture formation

We found 33 *L. lactis* strains among the 1232 strains tested that were able to enhance texture of both cow milk and soybean. To investigate if the texture of both matrices was dependent on the interactions between proteins and polysaccharides, we performed protein digestion before and during fermentation of the matrices using five strains with different texturing abilities. To investigate this, proteinase K was added to the matrices. The rationale for this was that proteinase K is a potent protease that indiscriminately cleaves proteins and that it would digest the matrix proteins into smaller polypeptide chains, and thus decrease the number of polysaccharide interaction sites per polypeptide chain. This should, therefore, lead to loss of texture. As can be seen in Figure S1 (Supporting Information), a loss of viscosity in all samples was observed when proteinase K was added (black) compared to the samples that were untreated (gray) or only added proteinase K buffer without proteinase K (striped). The observed drop in viscosity varies depending on the strain and this may be due to the different abilities of the polysaccharides to utilize proteins as network partners. This also indicates that there is a texturing component that is not protein-dependent, of which some or all could be due to the properties of the polysaccharides themselves. This result could explain why many of the same strains resulted in enhanced viscosity in the two matrices despite their different composition.

### Comparative genomics of polysaccharide biosynthesis in *L. Lactis*

Since enhanced texture is associated with the production of polysaccharides, mining for *eps* gene clusters was performed. A large diversity of gene clusters encoding the synthesis of exocellular polysaccharides via the Wzy-dependent pathway, including 10 novel *ep*s gene clusters, was found in the genomes of *L. lactis* (Figure S2, Supporting Information). Here, we used the nomenclature as described in (Poulsen et al. 2019).

Out of the 34 *L. lactis* strains able to enhance texture in milk, 22 strains contained the *eps* gene cluster with a high identity (99.95%–99.99% on the nucleotide level) to the *L. lactis cremoris* strain B40 (pNZ4000, GenBank AF036485): LC-10, LC-11, LC-15, LC-16, LC-17, LC-18, LC-19, LC-21, LC-22, LC-23, LC-24, LC-25, LC-26, LC-27, LC-28, LC-29, LC-30, LC-31, LC-32, LC-33, LC-34, and LC-35 (Table S1, Supporting Information). Novel *eps* gene cluster sequences were found in 10 texturing proprietary strains: LC-04, LC-05, LC-06, LC-07, LC-08, LC-09, LC-12, LC-13, LC-14, and LC-20 (Figure S2, Supporting Information). LC-07 and LC-04 have similar *eps* gene clusters, which might result in the same polysaccharide structure. The remaining texturing strains with novel *eps* gene clusters are presumably producing different types of polysaccharides, as they have variable glycosyltransferase genes along with different *wzy* and *wzx* genes.

Genes located at the 5' end of the *eps* gene cluster *epsRXABC*, which are involved in the modulation and assembly machinery of polysaccharide biosynthesis, as well as *epsL* and *lytR* at the 3' end, displayed the highest level of conservation (Figure S2, Supporting Information). The genes of the variable part including polymerase *wzy*, flippase *wzx*, and glucosyltransferases (GT) or other polymer-modifying enzymes, were mostly not similar between the strains, suggesting a large diversity within *eps* gene clusters, in agreement with what was observed previously (Poulsen et al. 2019, 2020). The common denominator for the texturing strains is that they all contain the genes required for polysaccharide production (Zeidan et al. 2017), such as *epsABCD-wzy-wzx* and several glycosyltransferases. Glycosyltransferases, together with other genes of the variable part, are potentially involved in the sequential building of the polysaccharide repeating unit, although their specific functions and underlying mode of action have not been established.

## Conclusion

The present work describes high-throughput screening for texturing *L. lactis* strains in cow’s milk and soybean drink and finds that many of the same strains are able to improve the texture in both matrices. We speculate that the same exocellular polysaccharides play a role in generating texture in both milk and soybean, despite the large compositional differences between the matrices, especially with regard to the structure of the proteins and the protein content. In both matrices proteins were important for texture formation, but also a nonprotein component influenced the viscosity, which very likely is the polysaccharides themselves. The findings indicate that it is the properties of the polysaccharides that drive the texture formation and determine if networks containing proteins can be formed. The nature of the interactions is challenging to investigate but is most likely of low-primary sequence specificity, i.e. the interaction is mediated through charged or hydrophobic clusters on the surface of the proteins possibly formed during the fermentation process.

## Supplementary Material

fnac117_Supplemental_File
