# Supplementary material for: Versatile Lactococcus lactis strains improve texture in both fermented milk and soybean matrices
Source: FEMS Microbiol Lett. 2022 Dec 1;369(1):fnac117. doi: 10.1093/femsle/fnac117 (PMC9772817; doi:10.1093/femsle/fnac117)
Supplement: fnac117_Supplemental_File [file fnac117_supplemental_file.pdf]

Supplementary Figure 1. Texture measurement (TADM area) of cow's milk and soybean samples fermented in 2-ml micro-titer plate scale with five different *L. lactis* strains (grey), in the presence of proteinase K (black) or protease buffer (striped).

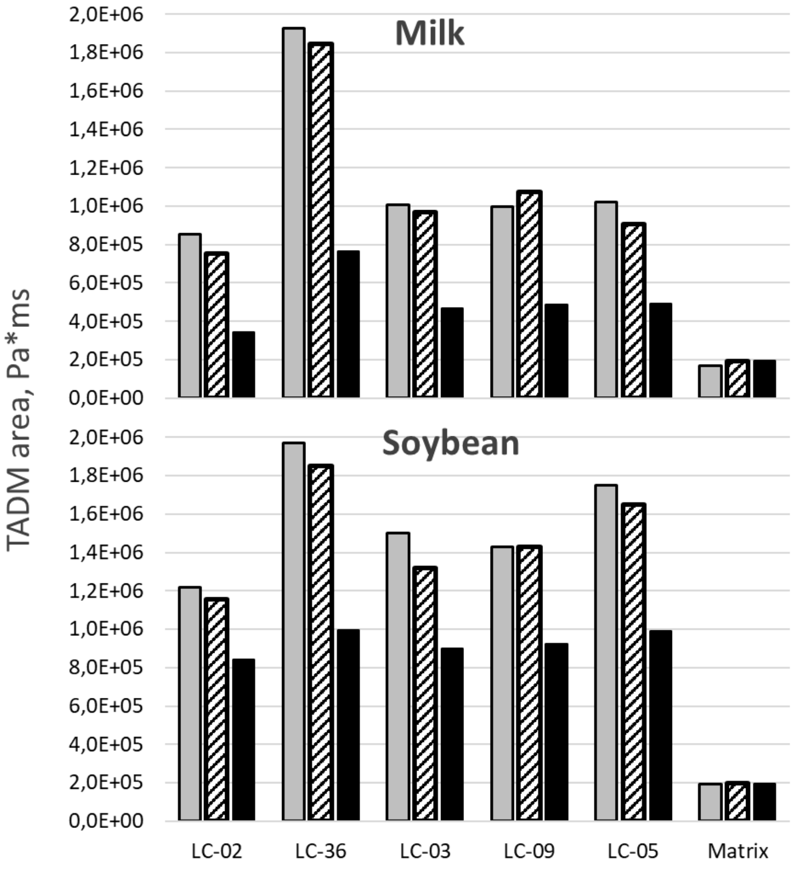

Supplementary Figure 2. Comparison of *eps* gene clusters of selected texturing *L. lactis* strains from the Chr. Hansen culture collection, and two publicly available *L. lactis* genomes, *L. lactis* subsp. *cremoris* NIZO B40 and *L. lactis* subsp. *lactis* KLDS 4.0325. Genes in the *eps* operon were categorized into groups based on the putative or established functions of their products as in (Zeidan et al. 2017; Poulsen et al 2019; Poulsen et al 2020). These include modulatory genes (yellow; phosphoregulatory module *epsABC*), polysaccharide assembly machinery genes (green; initiation *epsD*, polymerization *wzy*, export/flippase *wzx* and attachment *lytR*), genes encoding GT (orange; glucosyl transferases) necessary for the assembly of the repeating units, and genes encoding non-housekeeping functions (pink) required for the synthesis of activated sugar precursors and modification of the sugar residues. The functions of the three genes typically only present in the lactococcal *eps* gene clusters, *epsR*, *epsX* and *epsL*, remain to be elucidated. Mobile genetic elements are marked as IS. Genes with unknown functions or functions that might not be related to the polysaccharide production, *e.g.* mobile genetic elements, are in white, grey and blue. Grey boxes represent hypothetical proteins including proteins containing domains of unknown function, DUF1919, DUF1972 or DUF4422. Acetyltransferase-like ORF are represented by light blue boxes, while acyltransferase-like ORF, by dark blue boxes. Abbreviations: GT, glycosyltransferase; *wzy*, polymerase; *wzx*, flippase; NDP-sugar, nucleotide diphospho-sugar. All the genes are transcribed in one direction except for a few genes oriented in the opposite transcriptional sense, which are indicated with arrows.

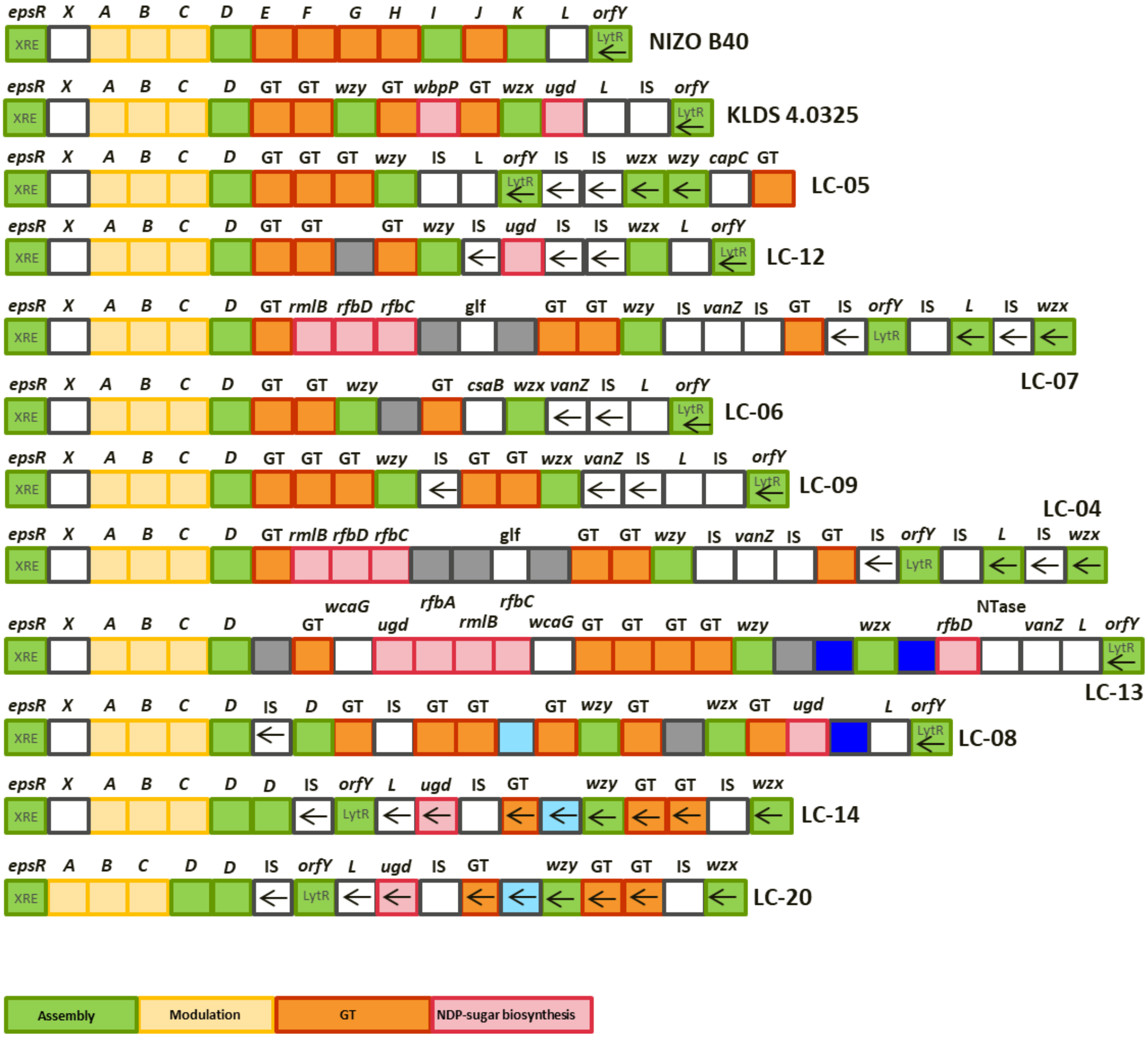

epsR X A B C D E F G H I J K L orfY
XRE LytR
NIZO B40
epsR X A B C D GT GT wzy GT wbpP GT wzx ugd L IS orfY
KLDS 4.0325
epsR X A B C D GT GT GT wzy IS L orfY IS IS wzx wzy capC GT
LC-05
epsR X A B C D GT GT GT wzy IS ugd IS IS wzx L orfY
LC-12
epsR X A B C D GT rmlB rfbD rfbC glf GT GT wzy IS vanZ IS GT IS orfY IS L IS wzx
LC-07
epsR X A B C D GT GT wzy GT csaB wzx vanZ IS L orfY
LC-06
epsR X A B C D GT GT GT wzy IS GT GT wzx vanZ IS L IS orfY
LC-09
epsR X A B C D GT rmlB rfbD rfbC glf GT GT wzy IS vanZ IS GT IS orfY IS L IS wzx
LC-04
epsR X A B C D wcaG GT ugd rfbA rmlB rfbC wcaG GT GT GT GT wzy wzx rfbD NTase vanZ L orfY
LC-13
epsR X A B C D IS D GT IS GT GT GT wzy GT wzx GT ugd L orfY
LC-08
epsR X A B C D D IS orfY L ugd IS GT wzy GT GT IS wzx
LC-14
epsR A B C D D IS orfY L ugd IS GT wzy GT GT IS wzx
LC-20
Assembly
Modulation
GT
NDP-sugar biosynthesis

Supplementary Table 1. Strains containing *eps* gene clusters with high identity to that of the *L. lactis* subsp. *cremoris* strain B40 (pNZ4000, GenBank AF036485). The level of identity on the nucleotide level is shown in %.

	**LC-19**	**LC-23**	**LC-10, LC-15, LC-11, LC-24**	**LC-18**	***B40***	**LC-16, LC-35, LC-29, LC-31, LC-28, LC-33, LC-32, LC-30, LC-26, LC-17, LC-27, LC-34, LC-21, LC-25, LC-22**
**LC-19**	100	99,95	99,95	99,95	99,95	99,96
**LC-23**	99,95	100	99,98	99,98	99,98	99,99
**LC-10, LC-15, LC-11, LC-24**	99,95	99,98	100	99,98	99,98	99,99
**LC-18**	99,95	99,98	99,98	100	99,98	99,99
B40	99,95	99,98	99,98	99,98	100	99,99
**LC-16, LC-35, LC-29, LC-31, LC-28, LC-33, LC-32, LC-30, LC-26, LC-17, LC-27, LC-34, LC-21, LC-25, LC-22**	99,96	99,99	99,99	99,99	99,99	100
